# Bioactive Compounds and Extracts from Traditional Herbs and Their Potential Anti-Inflammatory Health Effects

**DOI:** 10.3390/medicines5030076

**Published:** 2018-07-16

**Authors:** Antonio Serrano, Gaspar Ros, Gema Nieto

**Affiliations:** Department of Food Technology, Nutrition and Food Science, Veterinary Faculty University of Murcia, Campus de Espinardo, Espinardo, 30100 Murcia, Spain; antonio.serrano5@um.es (A.S.); gnieto@um.es (G.N.)

**Keywords:** anti-inflammatory, medicinal plants, chronic diseases, *Uncaria tomentosa*, *Harpagophytum procumbens*, *Myrciaria dubia*, *Ribes nigrum*, hesperidin

## Abstract

The inflammatory processes associated with several chronic illnesses like cardiovascular disease and cancer have been the focus of mechanistic studies of the pathogenicity of these diseases and of the use of different pharmacological and natural methods to prevent them. In this study we review the current evidence regarding the effectiveness of natural extracts from as-yet little-studied traditional botanical species in alleviating the inflammation process associated with several chronic diseases. Additionally, the intention is to expose the known pathways of action and the potential synergistic effects of the constituent compounds of the discussed extracts. It is noted that the here-studied extracts, which include black garlic rich in S-allylcystein, polyphenols from cat’s claw (*Uncaria tomentosa*), devil’s claw (*Harpagophytum procumbens*), camu-camu (*Myrciaria dubia*), and blackcurrant (*Ribes nigrum*), and citrus fruit extracts rich in hesperidin, have similar or greater effects than other, more extensively studied extracts such as tea and cocoa. The combined use of all of these extracts can give rise to synergetic effects with greater biological relevance at lower doses.

## 1. Introduction

Inflammation represents a biological response of the organism to a series of mechanical, chemical, or infectious stimuli. Its mission is to isolate, destroy, or dilute, as a form of localized protection. Inflammation can be chronic or acute depending on the characteristics of humoral response and the molecules involved. When inflammatory balance is altered, with excessive pro inflammatory signals (for example in the cyclooxygenase pathway), physiological damage can occur [1].

Chronic inflammation is a status derived from physiopathogenic situations such as metabolic syndrome or inflammatory bowel diseases that involve prolonged exposition to a number of potential pathogenic substances. Those substances are mainly inflammatory mediators like tumor necrosis factor alpha (TNF-α), and are linked to cancer initiation [2]. The combination of these factors leads to an unbalanced inflammatory status with an increment in markers like inflammatory cytokines, including TNF-α, interleukin (IL)-6, and IL-1β, which are also associated with cardiometabolic diseases [3].

Thus, a way to prevent inflammation which can lead to carcinogenesis or cardiovascular diseases is through the use of botanic extracts of spices and herbs which show both antioxidant and anti-inflammatory properties. For this reason, anti-inflammatory phytochemicals could represent an exogenous aid crucial for the prevention of chronic diseases mediated by inflammatory processes.

## 2. Natural Extracts and Compounds with Anti-Inflammatory Properties

### 2.1. Allium nigrum (Black Garlic)

**Figure 1:**
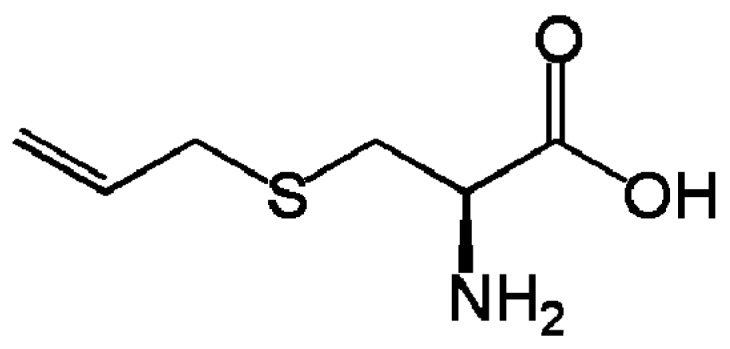
Chemical structure of S-allylcystein.

Garlic is a commonly used condiment with many biological activities due to its sulfur compounds [4] which have antioxidant and antimicrobial properties [5]. Black garlic is obtained through fermentation at a controlled high temperature (60–90 °C) and high humidity (80–90%). It has distinct bioactivity with respect to fresh garlic, conferring numerous benefits like anti-inflammatory, anticancer, and antiobesity activity [6]. This nutritional variation is characterized by a decrease in fructan, which is linked to an increase of fructose due to a Maillard reaction that subsequently impacts the color and taste [7]. Changes in the profile of volatile compounds provide black garlic with higher concentrations of S-alk(en)-yl-l-cysteine derivatives, while the quantity of ascorbic acid decreases due to thermal treatment [8]. Meanwhile, the concentrations of some flavonoids, pyruvate, total phenol, and the main antioxidant compound of black garlic, S-allylcystein, are increased [9] (Figure 1).

Some studies have isolated bioactive compounds from black garlic in order to evaluate their bioactivity. When black garlic aqueous extract is compared to raw garlic aqueous extract at similar dose range (between 31.25 μg/mL and 250 μg/mL), higher inhibition of tumor necrosis factor alpha (TNF-α) and prostaglandin E2 (PGE2) is reported for the aged garlic extract [10]. Kim et al. [11] demonstrated that even concentrations as low as between 5 μg/mL and 10 μg/mL of black garlic ethanol extract have anti-inflammatory effects as they inhibit nitric oxide (NO) and PGE2 production in lipopolysaccharide-stimulated (LPS-stimulated) RAW264.7 cells, and also decrease nitric oxide synthases and prostaglandin-endoperoxide synthase 2 expression. Choi et al. [12] showed that antioxidant power increased between day 0 and 21 of fermentation, reaching its limit at day 21 and keeping most of its properties at day 35 even though the levels of ascorbic acid were lower, as shown by Martínez-Casas et al. [8].

### 2.2. Uncaria tomentosa (“Cat’s Claw”)

**Figure 2:**
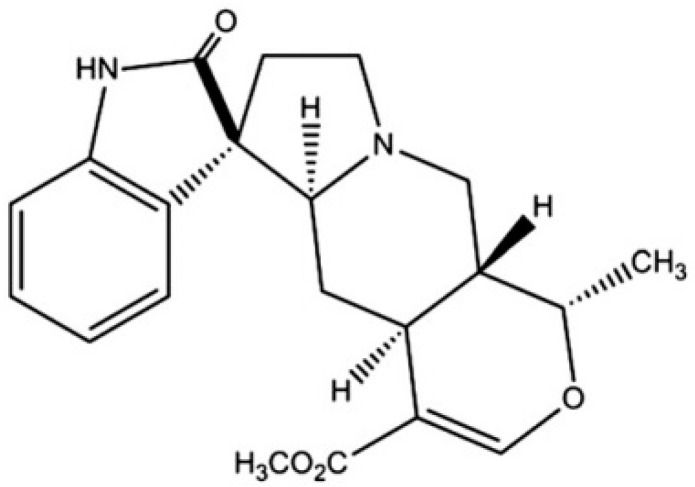
Chemical structure of mitraphylline.

*Uncaria tomentosa (*UT*)* is a climbing vine from Peru commonly known as cat’s claw from the Spanish “*Uña de Gato*” because of its thorns. It has been used over time in traditional medicine to treat diseases like rheumatism and cancer [13]. It has up to 32 phenolic compounds, including hydroxybenzoic acids, hydroxycinnamic acids, flavan-3-ols monomers, procyanidin dimers and trimers, flavalignans, and propelargonidin dimers [14]. One of the major bioactive compounds of UT is mitraphylline (Figure 2), which was isolated and evaluated by Rojas-Duran et al. [15] using a dose of 30 mg/kg/day for 3 days in mice, resulting in cytokine modulation providing fewer inflammatory signals.

The anticancer properties of UT are due to the synergistic activity of alkaloids with its non-polar compounds [16] like isopteropodine, pteropodine, isomitraphylline, uncarine F, and mitraphylline. These compounds also have exhibited anti-apoptotic properties in lymphoblastic leukemia [17]. The antioxidant properties of UT are attributed to its capacity to scavenge radicals such as superoxide anions and hydroxyl radicals, and also prevent lipid membrane oxidation [18]. Allen-Hall et al. [19] used human THP-1 monocytes (human monocytic cell line derived from an acute monocytic leukemia patient), reporting how UT affects the nuclear factor kappa-light-chain-enhancer of activated B cells (NF-κB) pathway, inhibiting inflammatory cytokines such as TNF-α and interleukin 1 beta (IL-1β). UT aqueous ethanol extract also has the potential to treat T-helper 1 immuno-mediated disorders with no cytotoxic or immunotoxicity at concentrations of 100 μg/mL and 500 μg/mL on murine splenocytes [20]

Another study suggested that both aqueous and alkaloid-enriched extract of UT act over the wnt-signaling pathway which influences degenerative diseases, diabetes, and cancer, modulating it to a less pathogenic environment [21].

### 2.3. Harpagophytum procumbens (Devil’s Claw)

*Harpagophytum procumbens* (HP), commonly named devil’s claw because of its fruits, is a perennial herbaceous plant which has roots that are traditionally used as anti-inflammatory agents for symptomatic treatment in arthritis and rheumatism. It grows mainly in the Kalahari Desert in the south of Africa. It had been linked with healthy properties like antimalarial, anticancer and uterotonic activities [22].

**Figure 3:**
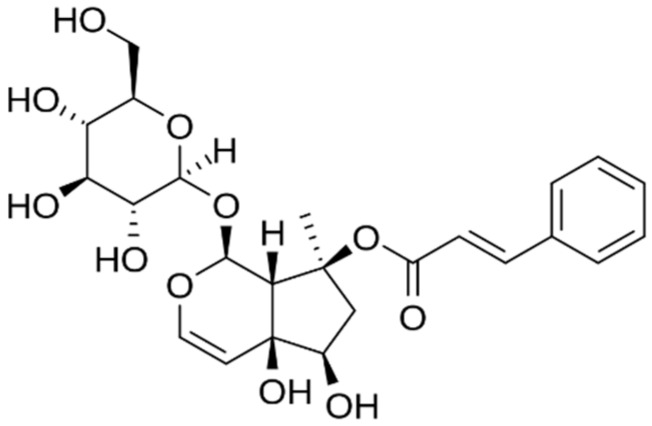
Chemical structure of harpagoside.

Its bioactivity is conferred by iridoids, a family of monoterpenoids. The glycoside fraction (containing mainly harpagoside; Figure 3) in devil’s claw has been proven to be antimutagenic against environmental injury [23].

In addition, anti-inflammatory properties have been widely evaluated. The extract of HP influences the synthesis and release of pro-inflammatory factors, inhibiting transcription factor activator protein 1 (AP-1) activity in murine macrophages and cytokine expression such as TNF-α and interleukin 6 (IL-6) when used at concentrations between 100 μg/mL and 200 μg/mL. It decreases COX-2 mRNA levels at concentrations between 50 μg/mL and 200 μg/mL [24]. In any case, devil’s claw’s effectiveness is not only based on its harpagosides but also on synergistic activity with other compounds, as seen by Hostanska et al. [25], who observed less cytokine TNF-α, IL-6, and interleukin 8 (IL-8) production at concentrations of 250 µg/mL of HP ethanolic extract.

Hapargophytum extract also exhibits the capacity to prevent oxidative stress or loss of cell viability against common oxidants [26]. The phytochemicals responsible for these effects are primarily verbascosides, followed by phenylethanoid-containing fractions from methanolic extract [27].

### 2.4. Myrciaria dubia (Camu Camu)

*Myrciaria dubia* (MD) is a shrub from the Amazon rainforest which produces a round red-colored berry with a strong acid flavor. It is characterized by its content in vitamin C and polyphenols which confer it antioxidant, anti-inflammatory, and antimicrobial activities [28]

Camu camu juice, due to its vitamin C content, has been demonstrated to have physiological antioxidant power, decreasing total reactive oxygen species and anti-inflammatory activity and reducing circulating C reactive protein in human at a dose of 70 mL/day [29]. However, polyphenols present in camu camu such as proanthocyanidins, ellagitannins, and ellagic acid derivatives also have antioxidant and anti-inflammatory activities [30]. Separately, higher antioxidant activities were reported for stachyurin and casuarinin together with other tannins present in the fruit [31]. Other authors focused on antigenotoxic effects linked to the antioxidant capacity of the compound mixture present in camu camu, demonstrating that regardless of whether the processes are acute, sub-acute, or chronic, the administration of juice at a concentration of 25%, 50%, and 100% by injection of 0.1 mL/10 g reduces significantly genotoxic damage induced by hydrogen peroxide in mice blood cells [32].

### 2.5. Citrus Fruits Rich in Hesperidin

**Figure 4:**
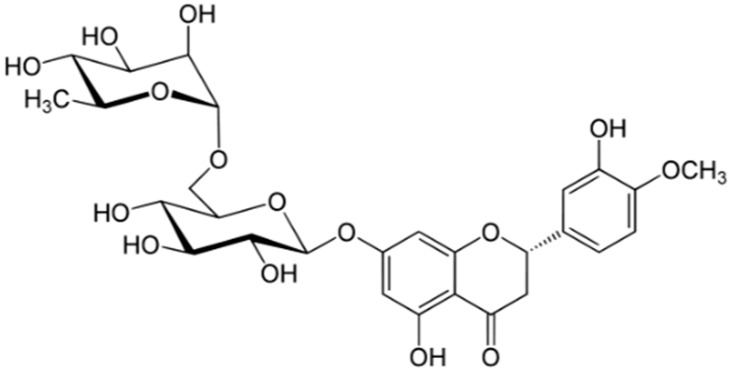
Chemical structure of hesperidin.

Hesperidin (Figure 4) is a flavanone from the flavonoid family and is mainly found in the epicarp, mesocarp, and endocarp of oranges, lemon, lime, and other citrus fruits [33].

Hesperidin has been widely studied due to its health benefits against cardiovascular diseases and cancer through its anti-inflammatory properties. It induces apoptotic cell death in gastric, colon, breast, lung, and liver cancer [34]. Its presence at a dose of 25 mg/kg a week before colon carcinogenesis induction and during the next 3 weeks in mice has been proven to enhance antioxidant-inhibiting reactive oxygen species and downregulate expression of inflammatory markers such as NF-κB, iNOS (a gene on chromosome 17q11.2-q12 that encodes inducible nitric oxide synthase), and COX-2 [35]. At a dose of 50 mg/kg 1 h before carrageenan injection it also exhibits antioxidative potential, enhancing endogenous antioxidants and decreasing inflammatory markers such as TNF-α, total leukocytes, neutrophils, lymphocytes, and nitrite concentrations [36]. Hesperidin’s anti-inflammatory properties have been observed in mice with induced cognitive impairment, showing that a pretreatment of 100 mg/kg to 200 mg/kg administered intraperitoneal once daily for 15 days modulates neuronal cell death, inhibiting the overexpression of inflammatory markers and providing neuroprotective effects [37].

Similar studies were conducted in rats. Those rats that were supplemented with doses of 50 mg/kg, 100 mg/kg, and 200 mg/kg prior to lipopolysaccharide-induced endotoxicity showed an improvement in endothelial status with respect to those that were not treated [38]. The capacity of hesperidin to down-regulate inflammatory status has been reported to improve rat postoperative ileus in a dose-dependent manner between 5 and 80 mg/kg [39]. A dose of 160 mg/kg in rats reduced lipid peroxidation and improved the activity of endogenous antioxidant enzymes, palliating effects of rheumatoid arthritis [40].

Clinical assays performed in 75 patients with myocardial infarction at a dose of 600 mg/day for four days showed an improvement in inflammatory markers and lipid profiles [41].

### 2.6. Ribes nigrum (Blackcurrant)

*Ribes nigrum* (RN) is a woody shrub natural from central and Eastern Europe. Its fruit, the blackcurrant, is traditionally used for the treatment of rheumatic disease. It contains significant concentrations of vitamin C and some polyphenols, mostly anthocyanins. As seen in other extracts, synergistic activities are key in natural extracts but in the blackcurrant, the most remarkable compounds are prodelphinidins [42]. Rutinosides and glucosides of delphinidin and cyaniding are the major compounds in blackcurrant extract, but also other compounds have also been found, such as myiricetin and quercentin glucosides, at lower concentrations [43]. Considering the presence of those compounds, it is not surprising that different studies found anti-inflammatory and antioxidant activities in blackcurrants.

Blackcurrant extract obtained from freeze-dried blackcurrants at a concentration of 1 mg/mL applied to cell culture supernatant down-regulates the expression of inflammatory mediators through the action of intestinal cells and macrophages stimulated with lipopolysaccharides [44]. Another study which also used RAW264.7 macrophages stimulated with LPS showed a reduction in mRNA levels of TNF-α, IL-1β, and iNOS when blackcurrant extract was added at a concentration of 0.2 mg/mL to cultured supernatant. [45]. Further studies with a mix of berries (blueberries, blackberries, and blackcurrants) support these results, using lower concentrations of between 10 and 20 µg/mL in cell cultures and obtaining lower levels of IL-1β messenger RNA [46].

Proanthocyanin-enriched blackcurrant extract suppressed IL-4 and IL-13 secretion from alveolar epithelial cells in a study performed with concentrations of 0.5–10 mg/mL total blackcurrant polyphenolic extract [47]. Concentrations of 5 µg/mL to 25 µg/mL of blackcurrant extract and cyanidin-3-O-glucoside on supernatant were used with monocyte-derived macrophages (U937), showing higher cell viability against nicotine and lower levels of IL-6 secretion when inflammation was induced through lipopolysaccharides [48].

Animal studies were also performed, obtaining anti-inflammatory results due to a depletion in the contents of TNF-α, IL-1β, IL-6, and IL-10 on Wistar rats pretreated with an intraperitoneal administration of proanthocyanidins from RN leaves at concentrations of 10 mg/kg, 30 mg/kg, 60 mg/kg, and 100 mg/kg [49]. In addition, the chemopreventive effects of anthocyanins from RN were tested against hepatic carcinogenesis in Sprague–Dawley rats using a dose between 100 mg/kg and 500 mg/kg of extract during four weeks, obtaining as a result antihepatocarcinogenic effects [50].

## 3. Comparing Emergent Extracts with Classics

There is a great complexity in the modes of action of the phytochemical species present in the extracts described above, and while most are used traditionally to palliate a broad spectrum of diseases, just a few natural extracts have been widely popularized.

One of these is green tea (*Camellia sinensis*) for which its phytocompounds have been proven to have anticancer properties [51]. It provides protection against environmental factors that affect homeostasis, such as genotoxic substances and free radicals [52]. Green coffee also exhibits a number of health benefits, such as protection of hepatic cells from oxidative damage [53]. Its chlorogenic acids inhibit LDL (low-density lipoprotein) peroxidation and COX-2 production, helping to prevent colon cancer and cardiovascular diseases [54], and down-regulate cytokines and proliferative factors [55]. Cocoa, from *Theobroma cacao*, has also been studied because of its antioxidant and anti-inflammatory characteristics [56]. The fruit from *Vitis vinifera*, commonly named grape, is also a source of antioxidant and anti-inflammatory compounds in both the seeds and the skin [57,58]. It is widely studied due to its involvement in wine production, which also gives rise to the presence of stilbenoids [59].

World famous foods such as tea, coffee, and cocoa have been studied extensively due to interest not only as foods that promote health but also for their commercial potential given high levels of production and exportation. For this reason, other botanical species potentially beneficial for health have been relegated to the background and should be studied more thoroughly

## 4. Conclusions

Nowadays, cardiovascular disease and different types of cancers represent major challenges to public health, and because identification is crucial. Growing interest in health can be an incentive when producing food and nutraceuticals that promote research in this field.

In this review, which includes a summary of the dose–response activity of selected extracts, we can see how different lesser-known extracts have an effectiveness similar to those of more widely used extracts. It is important to bear in mind that botanical species such as UT and HT at low doses have anti-inflammatory, anti-oxidant, and anti-carcinogenic effects when evaluated in cell and animal studies. Even species such as *Allium nigrum* are able to enhance their beneficial effects or add new ones after processing. Hesperidin has also been studied as a representative of citrus fruits given its anti-inflammatory and cardioprotective effects. To conclude, MD and RN suppose a source of antioxidants that are present in the fruit of these botanical species that can not only act as such but also enhance the beneficial effect of the extracts described above.

As we have seen in the selected extracts, synergistic activity is one of the factors that potentiates the effects of natural extracts against purified compounds. This way, it is assumable that the joint use of different sources of bioactive compounds could lead to higher effectiveness. However, the safety of these mixtures should be evaluated and the action pathways identified.
